# Smart Sensors for Smart Grid Reliability

**DOI:** 10.3390/s20082187

**Published:** 2020-04-13

**Authors:** Monica Alonso, Hortensia Amaris, Daniel Alcala, Diana M. Florez R.

**Affiliations:** 1 Electrical Engineering Department, Universidad Carlos III de Madrid, 28911 Leganés, Madrid, Spain; monica.alonso@uc3m.es; 2 Department Hydraulic, Universidad Politecnica de Madrid, 28040 Madrid, Spain; d.alcalag@upm.es; 3 Laboratoire d’Electrotechnique et d’Electronique de Puissance, Centrale Lille, Arts et Metiers Paristech, Universitey Lille, HEI, EA 2697 – L2EP, F-59000 Lille, France; diana.florez@yncrea.fr

**Keywords:** smart sensor, smart grid, reliability, adaptive protection, optimization

## Abstract

Sensors for monitoring electrical parameters over an entire electricity network infrastructure play a fundamental role in protecting smart grids and improving the network’s energy efficiency. When a short circuit takes place in a smart grid it has to be sensed as soon as possible to reduce its fault duration along the network and to reduce damage to the electricity infrastructure as well as personal injuries. Existing protection devices, which are used to sense the fault, range from classic analog electro-mechanics relays to modern intelligent electronic devices (IEDs). However, both types of devices have fixed adjustment settings (offline stage) and do not provide any coordination among them under real-time operation. In this paper, a new smart sensor is developed that offers the capability to update its adjustment settings during real-time operation, in coordination with the rest of the smart sensors spread over the network. The proposed sensor and the coordinated protection scheme were tested in a standard smart grid (IEEE 34-bus test system) under different short circuit scenarios and renewable energy penetration. Results suggest that the short-circuit fault sensed by the smart sensor is improved up to 80% and up to 64% compared with analog electromechanics relays and IEDs, respectively.

## 1. Introduction

In recent decades, power grids have been undergoing great changes led both by the adoption of new information and communication technologies (ICTs) and also by a massive integration of renewable energy sources. These modernized grids are commonly referred to as smart grids. The Smart Grids European Technology Platform defines a smart grid as “an electricity network that can intelligently integrate the actions of all users connected to it (generators, consumers, and those that do both) in order to efficiently deliver sustainable, economic, and secure electricity supply” [1]. Smart grids can monitor the state of a grid in real time and use the information to operate the grid in a secure, reliable, and stable way, with lower costs and improved energy efficiency [1,2,3]. In this context, sensors and actuators (network breakers) are crucial players in smart grid management [4,5,6]. Employing sensors for real-time monitoring allows one to deal with the power management of distributed energy resources, such as distributed generators (DGs) [7] and electric vehicles [8], as well as to improve the smart grid’s reliability [9].

Protection systems play an essential role in maintaining the promised power quality and reliability of smart grids. Grid reliability can be defined as the ability of a power network to perform its intended function of providing an adequate supply of electrical energy to customers efficiently with a reasonable assurance of continuity and quality [10]. Traditional reliability metrics have focused mainly on duration indices such as the System Average Interruption Duration Index (SAIDI) or frequency indexes such as the System Average Interruption Frequency Index (SAIFI), both measured on a yearly basis. SAIDI and SAIFI consider the number of customers affected by the outage and have a direct relationship with the performance of the protection devices. If the operation and tripping time of the protection devices are not optimally coordinated, a greater number of customers can be affected by the power interruption, consequently worsen the reliability indices. Some works have started to use new indices such as the actuator tripping time [11] or switching time [12], which are related to the operation of protection devices as shown in this paper.

The main objective of a protection system is to quickly detect an abnormal operation condition and decide on the most appropriate action to ensure that the faulted zone is isolated and the rest of the network works in a stable state. To detect a fault, such as a short-circuit, current monitoring devices are used to measure the current flow through the network. This current measurement is compared with a threshold current value or pick-up current of the protection devices to discriminate if the current is due to a fault situation or not. The protection unit calculates the operation time (tripping time) of the network actuators (breakers) according to their setting parameters. However, any malfunction in protection devices can result in significant damage to the electricity network infrastructure, a loss of electricity supply, and even endangerment of field crew.

Protection devices employed in distribution networks have evolved over time, from analog electromechanical relays, with reduced detection capabilities, to intelligent electronic devices (IEDs) that are able to transform the analog data gathered by current and voltage transducers into digital data and to provide adaptive protection functionalities remotely [13]. It must be noted that because of the limited processing capacity of IEDs, the updated protection setting parameters must be calculated by an external control center (centralized system) and communicated to the individual IEDs depending on the fault situation [13].

Distributed generators (DGs) such as photovoltaic installations and wind turbines are considered key actors for enabling the implementation of smart grids [14]. However, the protection of smart grids with DGs faces new challenges because of the stochastic behavior of renewable energy sources and their significant contribution to short-circuit currents when a fault is taking place. Until a few decades ago, a protection device’s settings were usually adjusted offline for the dominant or the most frequent topology of the power grid, taking into consideration the maximum load demand and the fixed energy production from the DG [15,16]. Nevertheless, the increasing integration of DG sources into distribution networks has revealed that classical protection schemes, using analog electromechanical relays, are not suitable for protecting smart grids in a safe and reliable way under high renewable energy penetration [17]. 

Some works in the literature have proposed the use of protection schemes intended for dealing with the distinctive features of smart grids. In [18] and [19], data gathered by several IEDs were used to improve the network fault location with the presence of DGs. In other cases, IEDs were provided with directional protection functionality where the adaptive protection settings were established by a control centre which communicated to the IEDs their setting parameters [20]. 

The protection schemes of smart grids must deal with all the changes in the distribution networks (such as topology and DG variability); therefore, maintaining fixed protection settings of protection devices is no longer valid [21]. To improve a smart grid’s reliability, new protection devices have to be designed with the following characteristics:

- They have to offer the ability to measure, detect, and automatically adapt their protection settings for each fault situation.
- These protection devices should be able to communicate both between one another and also with the actuators (network breakers) to share optimal settings as well as control actions in a decentralized architecture.
- Moreover, the design of new relay-based smart sensors, in which the coordination schemes could run inside each smart sensor, will help to speed up the fault detection and isolation process.

This paper presents a new relay-based smart sensor that is able to locally measure, detect, and isolate a short circuit (fault) in the minimum time possible. Furthermore, the smart sensor is able to update, locally, its setting parameters in real-time operation. Once a short circuit is detected, an adaptive protection scheme (APS) running inside each smart sensor optimizes its setting parameters in communication with the rest of the coordinated sensors that detect the fault. The optimal setting parameters are selected to minimize the opening duration time of the network breaker closest to the fault.

The smart sensors calculate the opening duration time of the network breakers, according to the fault measurements and their optimal settings, and they send those breakers the opening activation order. The information sharing among the coordinated smart sensors and the network breakers is based on the IEC 61850 Standard [22] using GOOSE messages.

The main contributions of this paper are twofold:

- A smart sensor is proposed that is able to locally calculate and update its parameters by means of an extended current-time search-space for each fault situation. Firstly, it should be noted that traditional protection devices use only fixed time-current curves according to [23] and secondly, the existing adaptive protection devices select one of the available time-current curves [23] in each fault condition. The smart sensor proposed in this paper is the only sensor capable of optimizing, directly, its protection setting parameters using the whole search-space made available by the protection instead of using the pre-defined time-current curves of the standard [23].
- A peer-to-peer communication is performed between coordinated sensors, based on the IEC 61850 [22] to optimize the operation time of the coordinated sensors.

It must be pointed out that, to the authors’ knowledge, none of these aspects have been addressed in the scientific literature.

## 2. Protection of Power Networks

The primary purpose of an electrical network is to efficiently deliver reliable electricity to consumers. Protection systems are of utmost importance in the reliability of electrical networks, since they are directly linked to interruptions in the power supply affecting the grid reliability (SAIFI, SAIDI). During network disturbances, such as short circuits or faults, protective devices used for the protection of the electrical network must detect the fault and activate the opening of the networks’ breakers in order to isolate the faulted zone. The correct intervention of the protective devices minimizes the outage time of the grid and reduces the effect of the fault currents on the network equipment. The fault-clearing process is based on the selectivity protection property [24], which means that only the protective devices that detect the fault activate the opening of the corresponding network breaker according to their detection setting parameters.

Analog electromechanical relays are characterized by inverse time-current curves defined by the IEC 60255-151 [23]. Electromechanical relays operate in a time that is inversely proportional to the fault current magnitude, (1):

(1) $$T_{r}=\frac{TDS\times \alpha }{{\left(\frac{I_{sc}}{I_{pickup}}\right)}^{n}-1}$$

where Tr represents the operating time of the electromechanical relays, $I_{sc}$ is the short-circuit current, $I_{pickup}$ is the current threshold value, and the time-dial setting (TDS) defines the time delay between the fault detection and the action of the corresponding breaker. The parameters α and n, listed in Table 1, are defined in the IEC 60255-151 standard [23] for different time-current relay characteristics (inverse, very inverse, extremely inverse).

To obtain a correct actuation of the protection equipment, power networks are subdivided into protection zones. Protection devices (relays) are responsible for detecting any fault that occurs in an assigned area, known as the primary protection zone. Moreover, they may properly provide backup protection to an area outside their primary zone (downstream). This means that each zone is protected by two relays: a primary relay (PR) and a backup relay (BR). The PR operates under short circuits inside its primary protection, and the breaker closest to the fault is the one that opens first, whereas the BR operates only if the PR fails to isolate the fault after a time delay. Each zone is overlapped by its adjacent zone to avoid unprotected (blind) areas. This overlapping is accomplished according to the location of the protection equipment electromechanical relays and network breakers.

**Figure 1:**
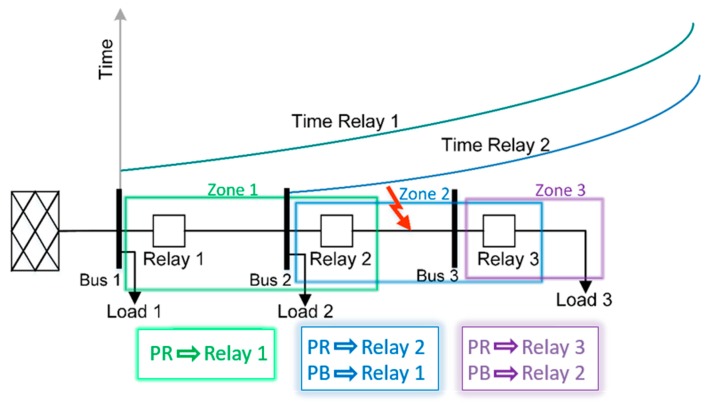
Typical current-time curves of a distribution feeder.

Electrical distribution networks typically consist of a secondary substation and a main feeder with lateral branches. In Figure 1, a radial distribution network, consisting of a secondary substation, a main feeder, three bus bars, and three loads, is used as an example to illustrate these principles. The placement of the three protective devices, namely, Relay 1, Relay 2, and Relay 3, delimits the three zones of protection; Zone 3 is protected by Relay 3 as its PR and by Relay 2 as its BR. Similarly, Relay 2 and Relay 1 are the PR and the BR, respectively, for Zone 2. Finally, Zone 1 has only Relay 1 which it is the PR. If a fault occurs in Zone 3 but the local PR of that zone (Relay 3) and the BR (Relay 2) fail to operate, then Relay 1 should operate to isolate the fault that occurred downstream of its protection zone. Figure 1 illustrates the typical relays’ operation curves and coordination time between their respective PRs and BRs, for a fault in Zone 2.

### Protection Challenges in Smart Grids

Smart grids are characterized by a high penetration of renewable energy sources, known as DGs, spread over a network. DG units are characterized by a bidirectional power flow from the DG to the grid. Depending on both the fault and the DG location, the operation of the protection devices can be affected. 

**Figure 2:**
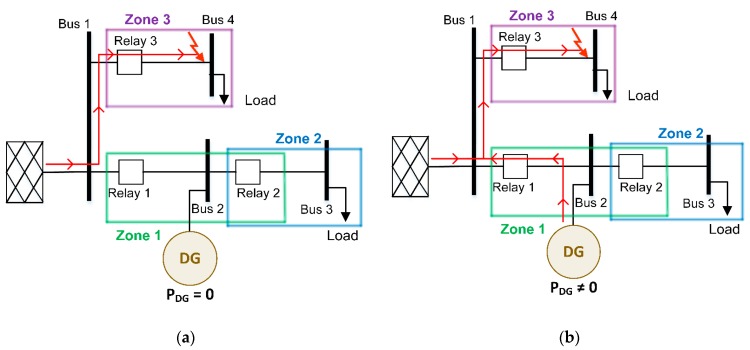
DG impact on fault current—sympathetic tripping: (**a**) without DG generation and (**b**) with large-scale DG generation.

Assume a radial distribution network with two feeders connected to the secondary substation (bus 1), similar to the one depicted in Figure 2. The distribution network has a DG unit, consisting of a photovoltaic (PV) farm, connected at Bus 2 and two customers located at Bus 3 and Bus 4. For protecting the grid, three relays are installed at Buses 1, 2, and 3 where Relay 1 is considered to be bidirectional. 

PV inverters are equipped with overcurrent protection that limits the inverter output current up to 1.3 of the rated current [25]. Depending on the inverter technology and the voltage drop at the PV location, they are required to remain connected to the grid providing LVRT capabilities during a period of time ranging from 20 ms to 200 ms [26,27,28]. Moreover, the IEEE Std 1547-2018 [26] states that the clearing time of protection DG units has to be selected from 0.16 s to 20 s depending on the voltage level at the DG connection point.

In this case, if a fault occurs in Zone 3, two different situations can unfold:

- If there are clouds or an absence of sunlight, then the net power injected by the PV generators into the grid is zero (Figure 2a). In this situation, the short-circuit current flows from the grid to the fault, and only Relay 3 is able to detect the fault and consequently isolate the faulted zone (Zone 3).
- In the case of large PV generators and diurnal conditions (Figure 2b), if a fault takes place in Zone 3 they have to remain connected to the grid up to 20 s after the short circuit feeding the fault from the DG to the grid. In this case, not only does Relay 3 detect the fault, but Relay 1 also detects a fault current contribution from the DG. In this situation, the current seen by the protective Relay 1 could exceed its threshold current (because of the short-circuit contribution from the large size PV generators) and may produce the unnecessary tripping of the healthy feeder before Relay 3 operates to clear the fault. This situation is called sympathetic tripping [29,30,31]. The sympathetic tripping effect does not happen for small PV generators because for low penetration levels, the effective limitation of the inverter current to below 1.3 times the rated current cannot provoke the tripping of Relay 1.

Given the previous issue, DG connection to smart grids causes a reverse power flow from the DG that could lead to the untimely action of the protection device, which is a selectivity problem. Therefore, traditional protection coordination schemes, with fixed setting parameters, become ineffective in smart grids with DGs. It is thus necessary to develop new protection schemes that are able to adapt the setting parameters of the protective devices in relation to the intermittent share of distributed generation [32,33].

Several approaches exist in the scientific literature for addressing the protection coordination issue in distribution networks with DGs. The authors in [34] proposed the use of dual setting directional relays based on current fault direction for directional protective devices (that is, IEDs) equipped with different time or current characteristic curves. Although protection settings are established using a nonlinear optimization function, they consider only the installed power of DGs, and the protection settings consequently remain fixed and are independent of any variation in generation or demand in real time. The work in [16] proposes a nonlinear optimization method for directional protective devices that are intended for protection planning purposes in future scenarios of DG growth. Although this methodology includes the requirement of protection setting adjustments for any new DG installation, it does not consider any adaptive, real-time adjustment of protection for variable grid topology, load or generation conditions. 

The previously mentioned protection schemes have one main limitation: the protection coordination problem considers a static network; that is, the adjustment of protective devices is achieved considering the amount of DG installed capacity in a predetermined network with a fixed demand. Therefore, for real-time variation in load demand or generation, the proposed schemes will only be valid for the conditions of generation, demand, and network topology for which the protective devices have been adjusted [15]. The evolution suffered by the electric power networks, as well as the growing concern about the improvement of their reliability, necessitates the development of new protection schemes to ameliorate the reaction time of protective devices to follow demand and generation variability [15,16,35,36]. 

## 3. Smart Sensors for Smart Grid Protection

### 3.1. Relay-Based Smart Sensor Architecture

In smart grids, several parameters must be measured, such as voltage, current, temperature, and phase, to be able to detect any parameter fluctuation in near real time and manage the corrective actions to assure grid reliability under fault conditions. Furthermore, the measured data must be readable by intelligent devices and contain a timestamp as well as the sensor location to facilitate decision support in smart grid operation [37].

**Figure 3:**
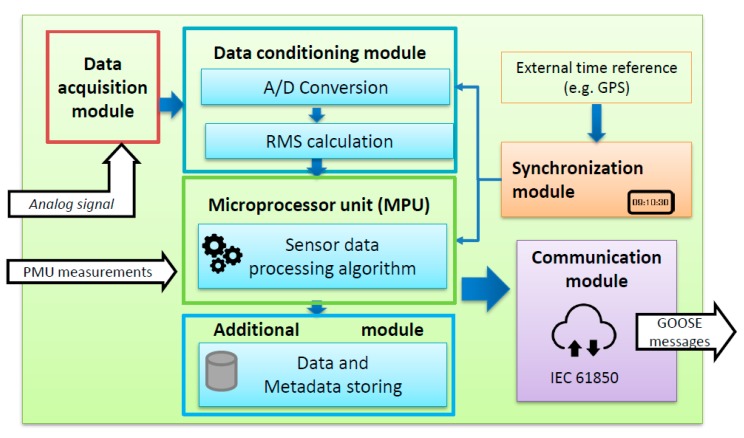
Structure of the relay-based smart sensor.

The relay-based smart sensor proposed in this paper is a single device composed of several modules (Figure 3):

- *Data acquisition module.* This module is in charge of monitoring and measuring the analogue signal in real time by means of the electricity transducers installed in the network.
- *Data conditioning*. In this module, the analogue electrical signal is conditioned and converted into a digital signal. After the sampling stage, the root mean square (RMS) value of the sampled signal is calculated and used to obtain the fundamental component of the measured signal.
- *Microprocessor unit (MPU)*. This module is responsible for processing the digital signal in order to detect and activate the network breaker to isolate the faulted zone. This is the core of the sensor in which the smart-sensor settings are updated depending on the network conditions and fault situation. Inside the MPU, the optimization algorithm (the APS) is responsible for selecting the optimal setting parameters that minimize the operation time of the PR and BR of the faulted zone. When a fault occurs, only the PR of the faulted zone executes the APS in the MPU to determine the optimal setting parameters of the primary and backup smart sensors. Moreover, the MPU algorithms are able to transform a physical measurement in an electric signal that could be processed, stored, and communicated to other devices through a bidirectional communication channel [1,37]. Finally, digital measured data could be stored internally on the device for future local or remote treatment.
- *Synchronization module*. An internal clock allows for the synchronization of the acquired digital data with an external time reference that could be shared with other devices, such as global positioning systems (GPS). Using measurement synchronization helps to improve the quality and accuracy of the measurement data. In smart grids, measurement synchronization is of great importance for operational and protection issues.
- *Communication module*. The communication module is responsible for the peer-to-peer communication between the smart sensors. For communication issues, smart sensors use the IEC 61850 standard [22]. When a fault takes place, the primary smart sensor communicates the optimal setting parameters to the backup smart sensor and activates the opening of the circuit breaker by GOOSE messages.
- *Additional module for metadata sensor storage*. This module allows the device to perform supplementary tasks related to the following: smart-sensor description and identification capability (self-description and self-identification); quality control of the work achieved by the smart sensor; and operation error reporting (self-diagnostics, self-testing and self-validation).

The operation carried out by the sensor is the following: 

A smart sensor “i^th^” measures the current seen by the sensor and calculates its RMS value. This RMS value is processed into the “i^th^”smart sensor’s MPU where it is compared with the I_pickup_ threshold value of the “i^th^” smart sensor. If the measured current value is higher than its I_pickup_ value it means that a fault has been sensed by the smart sensor “i^th^”. Consequently, the communication module of the smart sensor “i^th^” sends a GOOSE message to the downstream smart sensor “i^th+1^” which has to discriminate, itself, if the current seen by the sensor “i^th+1^” corresponds to a fault or not. If the sensor “i^th+1^” detects a fault it sends a goose message to the upstream sensor “i^th^” and to the downstream sensor “i^th+2^”. The peer-to-peer communication between consecutives sensors continues until it reaches the last smart sensor that does not sense any fault so that the faulted zone is identified. 

Once the faulted zone has been identified, the last two smart sensors that sensed the fault are identified as primary and backup sensors, respectively. The MPU of the primary smart sensor determines the optimal setting parameters of both smart sensors responsible for protecting the faulted zone, and the optimal setting parameters for the backup smart sensor are sent via GOOSE messages through the communication module. 

Afterwards, the primary sensor sends the opening order to the corresponding breaker via GOOSE messages by means of the communication module. If the primary sensor fails, the backup relay sends the opening order to the breaker according to its activation time value.

The smart sensor proposed in this paper offers added value to utilities, because of the telemetry functionality as well as remote operation and corrective action or predictive decision-making process, thereby improving grid efficiency and integrating new sensor technologies. 

The novelty of the smart sensor lies in the peer-to-peer communication capacity between the smart sensors of a faulted zone (the PR and BR) to isolate the fault and to establish a stable operation condition. The smart-sensor APS takes into account different smart grid operation conditions regarding DG integration and network topology. The APS also optimizes the primary and backup protection setting parameters to minimize the tripping time of the actuators (breakers) and isolate the faulted zone as quickly as possible. For that purpose, the APS employs three setting parameters (α, n, and TDS) that extend the operation area of the smart sensor beyond the standard curves defined by the IEC 60255-151 standard [23].

In the smart sensors, the IEC 61850 standard has been implemented so that the primary smart sensor (the PR) is able to communicate the adaptive protection setting parameters to the backup smart sensor (the BR) via GOOSE messages. 

The incorporation of these smart sensors into smart grid protection schemes allows for the enhancement of the network operation thanks to the real-time actions. It has to be highlighted that the smart sensor can be combined with other measurement devices such as phase measurement units (PMUs). PMUs have been used to monitor long transmission lines since 1970 to improve the network observability or to provide synchronized measurements to transmission line protection devices (differential protection, distance protection, frequency protection) [38,39]. PMUs can acquire fast sampling rate measurements which are sent to a central phase data concentrator to be processed, afterwards, by the control or protection network algorithms. They have been applied extensively in long transmission networks. However, their application to short-line distribution networks is still very limited. There is a study [40] that proposes the integration of PMUs with smart meters in distribution networks to improve the metering infrastructure. 

If PMUs were deployed in distribution networks as detailed in this paper, the proposed smart sensor can use, directly, the measurements provided by the installed PMUs. In this case, the PMU’s measurements will enter directly to the MPU where all the acquired measurements, provided by the conventional transducers and PMUs, will be processed to detect and activate the network breaker to isolate the faulted zone.

### 3.2. Relay-Based Smart Sensor Optimization Approach

The objective of the smart-sensor protection scheme is to minimize the operating time of the coordinated smart sensors (the PR and BR) while maintaining selectivity and enabling the isolation of the faulted zone. 

**Figure 4:**
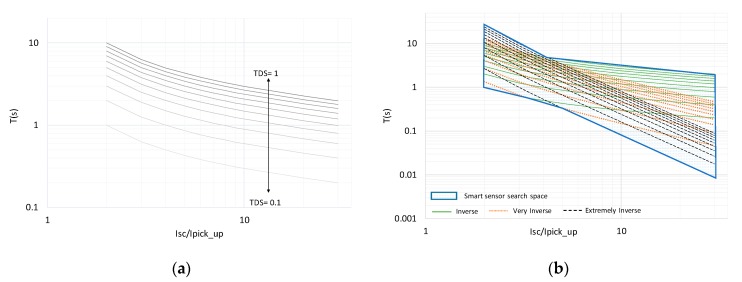
Time-current characteristics: (**a**) adaptive protection; (**b**) smart sensor.

As previously mentioned, classical analog electromechanical relays have proven to be inefficient when applied in networks with DGs [41]. To solve the problems of classical coordination, the authors in [42] employed IEDs with linear optimization techniques to adapt the protections settings using a centralized scheme. In this case, a constant ratio $(I_{sc}$/$I_{pickup})$ was considered, and a single variable in the optimization process was optimized (TDS). However, the results in [43] obtained from the linear optimization process using a single variable were not suitable because they do not consider all the network operating conditions (Figure 4a). To overcome these limitations, the protection coordination problem has been solved using IEDs with nonlinear optimization algorithms, in which TDS and the ratio $(I_{sc}$/$I_{pickup})$ were the decision variables [43]. A limitation of this approach is that it uses only the predefined time–current characteristic curves included in the IEC standard [23].

The proposed smart-sensor coordination approach is formulated as a nonlinear multivariable mixed-integer nonlinear programming (MINLP) optimization problem. Relay-based smart-sensor parameters α, n, and TDS are optimized by means of a genetic algorithm (GA) using three degrees of freedom in the coordination process. Moreover, the pickup currents are updated whenever a change in the distribution network is detected according to [44].

The advantage of optimizing three variables instead of two [45,46] is that the operating area of the smart sensor is expanded in the available search space, as illustrated in Figure 4b; this overcomes the restrictions imposed by the predefined time–current curves in [23]. This aspect has not been addressed before in the scientific literature and is of utmost relevance to improve the reliability of the smart grid.

Note that for real-time operation, where only two relay-based smart sensors must be coordinated for each zone, the optimization of three degrees of freedom for each smart sensor does not require a relevant computational burden.

### 3.3. Relay-Based Smart Sensor Communication

A crucial aspect in smart grids is the communication between devices connected to the network. Several standards have emerged as alternatives to create the backbone and communications network for smart grids. The most highlighted outcome of this standardization initiative is the IEC 61850 standard because of its ability to provide standard object-oriented modeling that could be mapped to various protocols. The IEC 61850 [22] is an international standard for the automation of measurement, control, and protection of secondary substations in distribution networks. It establishes a data model and a communication architecture that enable the interoperability of distribution networks by turning physical devices into logical devices. 

GOOSE messages entail a fast communication channel to share events-related information between multiple smart grid devices mapped by the IEC 61850, mainly those related to the critical information of smart grids. Furthermore, GOOSE messaging allows for the exchange of both digital and analogue data between peer-to-peer devices with an average processing rate of 12 ms. Peer-to-peer communication in the IEC 61850 environment introduces the concept of publisher and subscriber devices regarding the information exchange. When a device publishes a GOOSE message, only subscriber devices receive it and react according to their functionality in the distribution network. 

In this paper, IEC 61850 and GOOSE messaging are used with the aim of sharing the optimal protection setting parameters between the PR and BR of a faulted zone and sharing the tripping signal to the network breaker to isolate the fault. For that purpose, the primary smart sensor runs the APS and publishes the optimal protection setting parameters to the BR of the faulted zone and the associated tripping time of the network breaker to the primary smart relay. Although the GOOSE messaging information is published for all devices connected to the distribution network, only subscriber devices (backup smart sensor and primary network breaker) receive it. 

**Figure 5:**
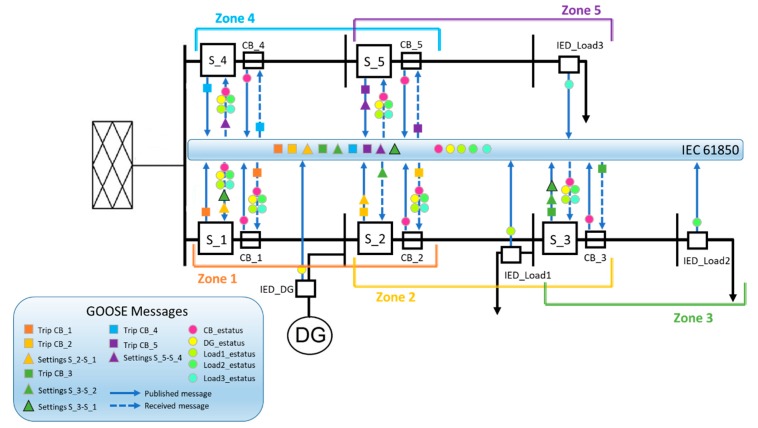
APS exchange information: smart-sensor coordination.

Figure 5 illustrates the communication via GOOSE messages among the different elements involved in the optimal coordination of the smart sensors of a faulted zone. When a fault occurs in the smart grid, the APS—hosted in the MPU of the primary smart sensors of the faulted zone—determines the optimal setting parameters of the pair of smart sensors to be coordinated. The adjustment parameters of the backup smart sensor are then sent by publishing a GOOSE message that is received only by the backup smart sensor of the faulted zone. Once the parameters (α, n, and TDS) of the primary and backup smart sensors have been updated according to the faulted operation condition, smart sensors determine the actuators’ activation time ($t_{rp}\;and\;t_{rb})$ and send a GOOSE message with the trip request to the respective network breaker. 

## 4. Smart-Sensor Adaptive Protection Coordination Scheme (APS)

### 4.1. Objective Function

To determine the opening orders of network breakers, the relay-based smart sensors’ operating time ($T_{r}$) is computed as a function of TDS and parameters *α* and *n* using Equation (2). These parameters are updated according to each situation of grid topology (“t”), fault type (“c”), faulted zone (“z”)b and DG penetration level (“d”) (3).

(2) $$T_{r\_tczd}=f\left(TDS_{r\_tczd},\;\alpha_{r\_tczd},\;n_{r\_tczd}\right)$$

(3) $$T_{r,tczd}=TDS_{r\_tczd}\times \frac{\alpha_{r\_tczd}}{{\left(\frac{{I_{sc}}_{r\_tczd}}{{I_{pickup}}_{td}}\right)}^{n_{r\_tczd}}-1}$$

The coordination of relay-based smart sensors is formulated as an MINLP optimization problem; see Equation (4):

(4) $$Min\;T_{operation}=W_{i}\overset{m}{\underset{i=1}{\sum }}T_{rp\_tcdz}^{}+W_{j}\overset{n}{\underset{j=1}{\sum }}\Delta T_{rp,rb\_tcdz}^{}$$

where:

- $W_{i}$ and $W_{j}\;$ are the weights assigned to the activation times of the primary smart sensors $r_{p}$ and the backup smart sensors $r_{b}$;
- $T_{rp\_tcdz}^{}$ is the activation time of the primary smart sensor $r_{p}$; and
- $\Delta T_{rp,rb\_tcdz}^{}$ is the coordination time between the primary smart sensor $r_{p}$ and the backup smart sensor $r_{b}$, (5), (6).

(5) $$\;T_{rp\_tcdz}-T_{rb\_tcdz}\geq CTI_{rp,rb}$$

(6) $$\Delta T_{rp,rb\_tcdz}^{}=TDS_{rp\_tcdz}\times \frac{\alpha_{rp\_tcdz}}{{\left(\frac{{I_{sc}}_{rp\_tcdz}}{{I_{pickup}}_{td}}\right)}^{n_{rp\_tcdz}}-1}-TDS_{rb\_tcdz}\times \frac{\alpha_{rb\_tcdz}}{{\left(\frac{{I_{sc}}_{rb\_tcdz}}{{I_{pickup}}_{td}}\right)}^{n_{rb\_tcdz}}-1}-CTI_{rp,rb}$$

### 4.2. Constraints

The proposed optimization problem is subjected to the following constraints:

#### 4.2.1. Coordination Criterion

The protection coordination criterion establishes the minimum time elapsed between the operation of the primary and the backup protection, known as the coordination time interval (CTI). To ensure the correct coordination between the primary and backup smart sensors, the coordination time must be greater than the minimum established time (7):

(7) $$CTI_{rp,rb_{min}}\leq CTI_{rp,rb}$$

#### 4.2.2. Operation Time Limits

The time that a smart sensor takes to detect a fault produced in its zone of influence and activate the opening of the breaker must be bounded. Equation (8) determines the operating time limits of the relay-based smart sensor:

(8) $$t_{r,max}\leq t_{r\_tcdz}\leq t_{r,min}$$

where $\;t_{r,min}$ and $t_{r,max}\;$ are the minimum and the maximum response times of the smart sensor r, respectively, for a fault in its operation area *n*.

#### 4.2.3. Time-Dial Setting (TDS) Limits

The operating time of the smart sensor is directly proportional to the TDS. Therefore, the smart sensor’s operating time is restricted by the limit values of the TDS (9).

(9) $$TDS_{r,max}\leq TDS_{r\_tcdz}\leq TDS_{r,min}$$

where $TDS_{r,min}$ and $TDS_{r,max}$ are the minimum and the maximum TDS values for the smart sensor *r*, respectively.

#### 4.2.4. Plug Setting Multiplier (PSM) Limits 

The plug setting multiplier (PSM) is the ratio of the fault current ($I_{sc}$) in the smart sensor to its pickup current ($I_{pickup}$). The limits established for the PSM are expressed in (10).

(10) $$PSM_{r,max}\leq PSM_{r}\leq PSM_{r,min}$$

where $PSM_{r,min}$ and $PSM_{r,max}$ are the minimum and the maximum PSM values for the smart sensor *r*, respectively.

#### 4.2.5. Relay-Based Smart Sensor’s Operating Characteristics

To extend the search space of the relay-based smart sensor, the smart sensor parameters α and n are optimized considering the maximum and the minimum values established by [23] for each smart sensor as expressed in (11) and (12), respectively:

(11) $$\alpha_{r,min}\leq \alpha_{r\_tcn}\leq \alpha_{r,max}$$

(12) $$n_{r,min}\leq n_{n\_tcn}\leq n_{n,max}$$

To solve the convex problem, an MINLP method is employed and solved by a GA. 

After the execution of the APS in the primary smart sensors of the faulted zone, the optimal characteristic parameters of these smart sensors are updated ($\alpha_{rp},\;n_{rp},\;and\;TDS_{rp}$), and the ones corresponding to the BR are sent via GOOSE messages ($\alpha_{rb},\;n_{rb},\;and\;TDS_{rb}$). Once the setting parameters are updated in the primary and secondary smart sensors, the activation time of both relay-based smart sensors is computed. The trip signal of the network breaker associated with the primary smart sensor is sent immediately via GOOSE messages. However, if the fault is not isolated by the primary smart sensor, then the trip signal computed by the backup smart sensor is sent to its actuator. 

### 4.3. Genetic Algorithm Process

The programming of the relay-based smart-sensor optimization coordination was carried out by means of a GA. Genetic algorithms belong to the meta-heuristic algorithms which have proven to be suitable for global search optimization techniques, dealing with linear and nonlinear, continuous or discontinuous as well as convex problems [47,48,49,50]. GA is a multipoint and population-based search methodology, so that the possibility of finding a global optimum solution is higher than that of other optimization methods, such as single-point search methodology, due to the possibility to explore the search space in different directions simultaneously [51,52].

Among other heuristic algorithms, Tabu search and Ant Colony are time-consuming methods; by contrast, GA computes rapidly [53]. Simulated annealing and neural networks present slow convergence and could be trapped in a local minimum, while GA has the ability to perform a global search [53].

**Figure 6:**
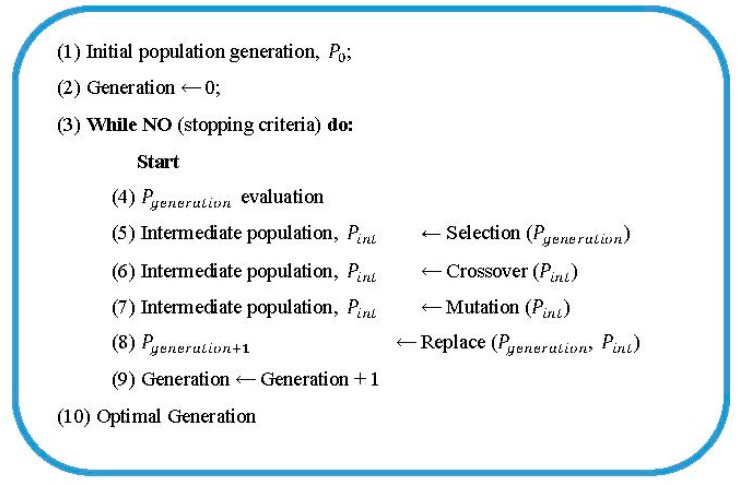
GA pseudocode.

The genetic algorithm pseudocode is presented in Figure 6. The steps of the GA programming are described below.

#### 4.3.1. Encoding Solutions (Step 1)

The first step is the determination of the method for coding the potential solutions of the problem, namely, defining the encoding of the chromosomes used in the GA.

The variables to be encoded are α, n, and TDS. These three variables used in the smart-sensor coordination process are considered integer numbers. Therefore, the chromosome used by the GA is composed of integer variables and its size depends on the number of smart sensors that have to be simultaneously coordinated. For each relay-based smart sensor ($N_{s}$) to coordinate in the power network, three genes ($2*N_{S}$) are needed, representing the variables α, n, and TDS of each relay-based smart sensor. The size of the chromosomes used will thus follow the expression $3*N_{s}$. Table 2 displays the chromosome structure used in the GA.

#### 4.3.2. Initial Population (Step 2)

The initial population of the problem is generated by a random procedure in which the variables’ constraints are taken into account: the limit values of α, n, and TDS.

The population size should be large enough to guarantee an optimal exploration of the solution space.

#### 4.3.3. Genetic Operators (Steps 3 to 9)

From this step (Step 3), and until the stop criteria are met, a loop begins whose objective is to generate a new population, $P_{generation+1}$, by applying genetic operators to the current population, $P_{generation}$.

Once the objective function has been evaluated (F (y), Step 4), and as long as the stop criteria are not met, the genetic operators are applied to create the next study population by first selecting (Step 5) the individuals that will constitute the parents in the reproductive process. The GA uses the roulette selection method to prioritize solutions that yield better aptitude values over those obtaining worse aptitude values. This method allows the best search areas to be exploited.

After the population of parents is obtained, the crossing is carried out (Step 6). Among all the techniques related to this process, the one chosen here is the single point crossing technique, since the size of the chromosomes used is small. Moreover, the utilization of other techniques could break the chain of the population considerably, leading to a loss of relevant information about the improved search areas. For the crossing probability, a value of 0.8 has been established (i.e., applying a crossing over 80% of the population).

The next step in the generation process of a new population is the application of the mutation operator (Step 7). The objective of this operator is to introduce diversity in the population or rescue information that may have been lost in the selection and crossing processes. Since the probability of crossover is high, a mutation probability ($p_{m}=0.1\%)$ has been selected that allows for the expansion of the exploratory capacity of the algorithm.

Finally, it is necessary to introduce children into the existing population to generate the population employed in the following iterative process. The option of replacement (Step 8) of the parents with the children has been chosen, based on the theory of evolution, according to which the children inherit the best properties of the parents in terms of adaptation to the environment.

From the intermediate population, composed of children resulting from the crossing and mutation processes and along with the population of the current generation, a new population corresponding to the next generation, $P_{generation+1}$, is obtained.

#### 4.3.4. Stopping Criteria

Two stop criteria have been defined:

- Tolerance of solutions: of the suitable values obtained by the best solution of two consecutive generations, the minimum tolerance value considered is 10^−6^.
- Maximum number of generations: a maximum number of 100 generations has been chosen.

In this paper, the number of smart sensors to coordinate simultaneously is two (N_s_ = 2) for each fault situation (PR, BR). The genetic algorithm is in charge of optimizing the tripping time of two consecutive relays (PR and BR) once the fault has been detected. Consequently, it can be easily scaled up to large network because, independently of the network size, the genetic algorithm optimizes only the parameters for the primary and backup relay where the fault has been detected.

## 5. Case studies

### 5.1. Power Network Description 

**Figure 7:**
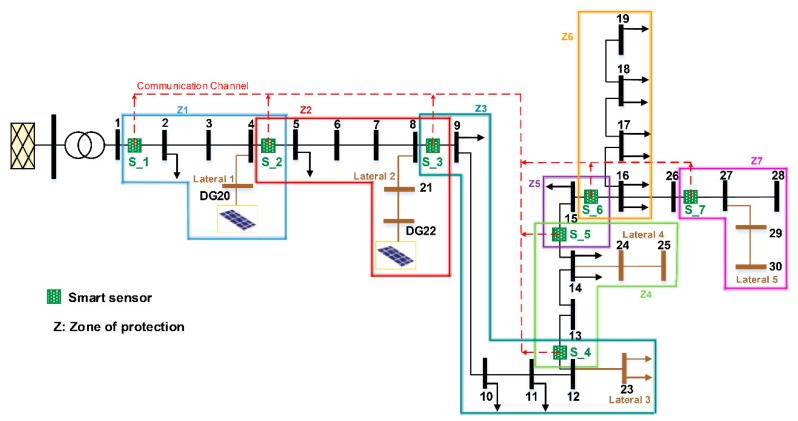
Modified IEEE 34-Node Test Feeder.

The proposed smart-sensor coordination scheme was implemented in a smart grid based on the topology of the IEEE 34-Node Test Feeder [54], as illustrated in Figure 7. The network consists of the following:

- a secondary substation (69 kV/24.9 kV);
- a main feeder and five laterals;
- two photovoltaic PV farms at Nodes 20 (DG20) and 22 (DG22) with a rated power of 12 MW;
- nineteen customers with a nominal load demand of 12 MW; and
- seven relay-based smart sensors (S_1 to S_7) at Nodes 1, 4, 8, 12, 14, and 16 of the main feeder and at Node 26 of Lateral 5.

The smart sensors’ location sectionalizes the main feeder of the studied smart grid into seven protection zones (Table 3). The lengths of the medium voltage power lines range from 85.3 m to 14.7 km [54]. Smart sensors are spread along the main feeder with a separation distance ranging from 2.4 km (zone 5) to 17.7 km (zone 3). Data regarding the distribution power network are provided in references [44,54]. Each relay-based smart sensor hosts the APS in its MPU in order to determine the optimal setting parameters when a fault is detected in its primary protective zone and to calculate the activation time of network breakers. 

### 5.2. Operation of the Proposed Smart-Sensor Coordination 

Seven fault scenarios were considered to test the accuracy of the proposed smart-sensor coordination (see Table 4). Scenarios A, B, and C correspond to a three-phase fault, and scenarios D and E correspond to a single-phase-to-ground fault in the main line of the distribution network with different DG conditions. In Scenario F, a single-phase fault in a lateral (Lateral 5) was analyzed.

Scenario B was chosen to demonstrate the application of the protective smart protection scheme. In this scenario (Table 4), a three-phase fault appears at the main feeder between buses 6 and 7, inside Protection Zone 2. The PR and BR of this protection zone correspond to Smart Sensor 2 (S_2) and Smart Sensor 1 (S_1), respectively. In Scenario B, the only available DG is the PV-generation unit connected at bus 22 (DG22), with a penetration level of 50%, which corresponds to 4.94 MW. The total power network demand at the fault instant is 80% of the substation transformer capacity, which is 9.88 MW. 

The operation of the smart-sensor coordination is explained as follows:

- Once the fault is detected, the PR-based smart sensor (S_2) runs the APS hosted in its MPU in order to determine the optimal setting parameters of both S_2 and S_1 for the optimal isolation of the faulted zone (Z2). The APS employs the extended area of the relay-based smart sensors to be coordinated, delimited by the imposed limits in (11) and (12) and the pick-up current values given by [44]. The optimization algorithm uses these values to determine the optimal parameters α, n, and TDS, which yield the minimum operation time of the PR- and BR-based smart sensors. Table 5 lists the optimal α, n, and TDS values for the pair of relay-based smart sensors of the faulted Zone 2 (S_1, S_2 in Scenario B) given by the execution of the GA in the APS.
- After calculation of the optimal setting parameters of the relay-based smart sensors by the APS, the smart sensors compute the activation time of the actuator in charge of opening and clearing this faulted zone. According to S_1 and S_2′s setting parameters in Table 5, the primary (S_2) and backup (S_1) tripping times are 0.1 and 0.29 s, respectively. The tripping time is sent via GOOSE messages to the network breakers associated with S_2. The network breaker associated with S_1 only acts if Network Breaker 2 fails to isolate the faulted zone.

### 5.3. Comparison with Analogue Electromechanical Relays and IEDs 

To demonstrate the network reliability improvement by the smart sensors, the tripping times of three protection methods are compared: the classic coordination, a linear programming coordination, and the proposed optimal smart-sensor approach. The protective devices are electromechanical relays (ERs) when the classical protection schemes are implemented, IEDs in case of the linear programming function, and relay-based smart sensors (S_1 and S_2) for the proposed optimal approach. In all cases, fault Scenario B is considered (three-phase fault in Z2).

**Figure 8:**
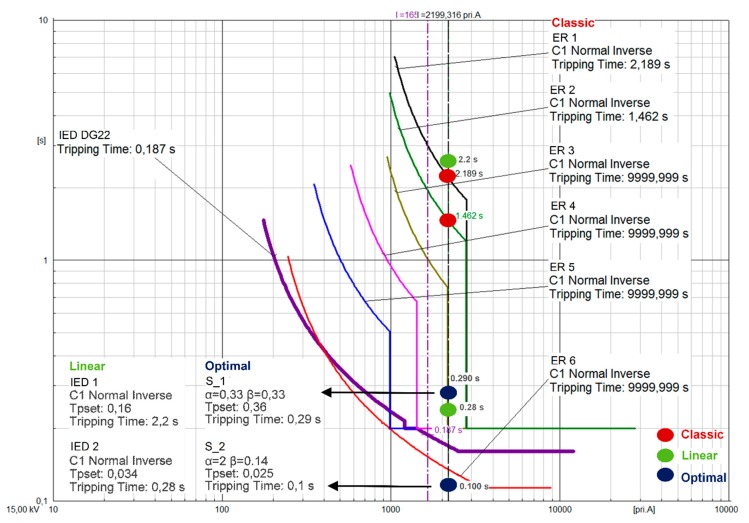
Tripping time comparison of the three protection schemes (classical, linear, and optimal smart-sensor coordination) for Scenario B.

Figure 8 depicts the activation times for the three protection schemes. In Figure 8,

- the red dots indicate the tripping times of ER1 and ER2 when the classical tripping function is considered according to IEC standard 60255-151 [23,24];
- the green dots indicate the tripping times for IED1 and IED2 when the linear programming technique is implemented [40]; and
- the blue dots indicate the optimal tripping time established by the smart-sensor optimal APS coordination (S_1 and S_2).

As illustrated in Figure 8, the APS formulation proposed in this paper, hosted in the smart sensors’ MPU, is able to reduce the fault activation time (tripping time) compared to classical electromechanical relays and IEDs with linear protection schemes by up to 80% and 64%, respectively. One of the advantages of the genetic algorithm is that it allows us to calculate the opening orders of network breakers with the minimum time. As illustrated in Figure 8, the opening orders of network breakers for the PR and BR are 0.1 s and 0.29 s, respectively. These times increase for the linear optimization (IEDs), up to 0.28 s for the PR and 2.2 s for the BR. In the case of the classic coordination with ER, the opening order of PR is 1.46 s, and 2.189 s for the BR. It can be deduced that the GA is the only algorithm that is able to reduce the tripping times of the PR and BR and consequently, the opening order of the breaker in the faulted section. Compared with other optimization functions, the proposed smart sensor coordination establishes a tripping time lower than that proposed in [55] which ranges from (0.21–0.27) s for the primary relay and from (0.5–0.75) s for the backup relay.

The genetic algorithm was programmed in Matlab^®^ [56] on a 2.7 GHz Intel Core i5-5200 with 8 GB of RAM. The average computation time used for optimizing the tripping time of both sensors (S_1, S_2) was 0.7 s, which is lower than the 2 min detailed in [57].

IEC 61850-5 states that the transfer time of GOOSE messaging for a Trip command shall be such that the command should arrive at the destination device within 3 ms [58]; consequently, the effective communication between the PR and BR is less than 3 ms. Moreover, as illustrated in Figure 8, the opening orders of network breakers for the PR and BR are 0.1 s and 0.29 s, respectively. It has to be noted that the PR is located next to the breaker actuator. Once the breaker receives the opening order it will need an average time of 48 ms [59] to disconnect the faulted zone. It can be deduced that the relation between the time of effective communication between the sensors and the time of disconnection of the short circuit in the network is inferior to 1% for Scenario B.

### 5.4. Influence of Different Fault Types and Fault Locations

The proposed smart-sensor optimal protection coordination is now examined for different fault conditions:

- Fault type: a three-phase fault (Scenarios A and C) and a single-phase-to-ground fault (Scenarios D and E).
- Fault location: a fault in Zone 2 of the main feeder (Scenario E) and a fault in Zone 7 corresponding to Lateral 5 (Scenario F).
- Operation conditions: one or two DG units in operation in the smart grid. In both cases, the DG penetration level is 50%.

**Figure 9:**
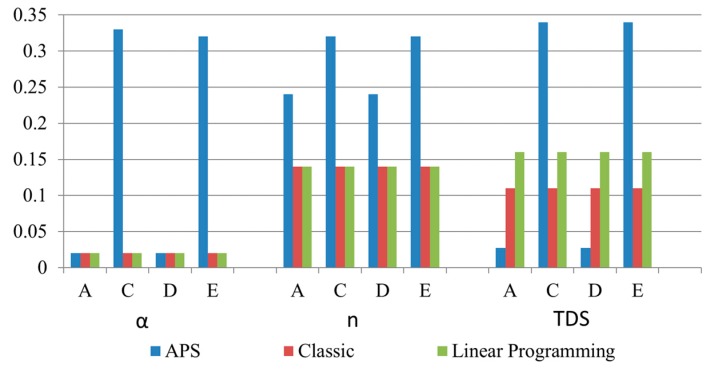
Comparison of backup relay settings of the three protection schemes (classical, linear, and optimal smart-sensor coordination) for different fault types.

Figure 9 illustrates, for the main feeder fault scenarios (A, C, D and E), the optimal settings of parameters α, n, and TDS for the backup smart sensor for the coordination of three different protective devices: an ER with IEC overcurrent protection curves (classic) [24], an IED with the adjustment of setting parameters by linear programming (lineal) [40], and smart sensors with optimal protection settings given by the APS of the primary smart sensor and setting parameter communication via GOOSE messages. As can be seen from Figure 9, the smart sensor is the only protection device capable of adapting the setting values of the backup smart sensor for different fault types. In the case of the primary smart sensor and for Scenarios A, C, D, and E, the primary smart sensor’s APS sets the parameters of the primary smart sensor to α = 0.02, n = 0.14, and TDS = 0.16. These optimum settings correspond to an operating tripping time less than 0.1 s for the primary smart sensor.

The results of the operating times obtained by the three compared coordination methods (classical, linear programming, and APS) for the main feeder faults as well as the lateral fault (Scenarios A, C, D, E, and F) are presented in Table 6. As observed, the values established by the APS hosted in the smart sensors’ MPU are substantially lower than those established by classical and linear coordination methods.

### 5.5. Influence of DG Penetration Levels

To validate the smart-sensor protection coordination proposed in this paper under different network situations, two important situations are studied: (i) the influence of DG penetration levels and (ii) the influence of the impedance fault values. High impedance faults in distribution networks produce low-magnitude fault currents that could be considered as normal current values corresponding to normal operations. 

**Figure 10:**
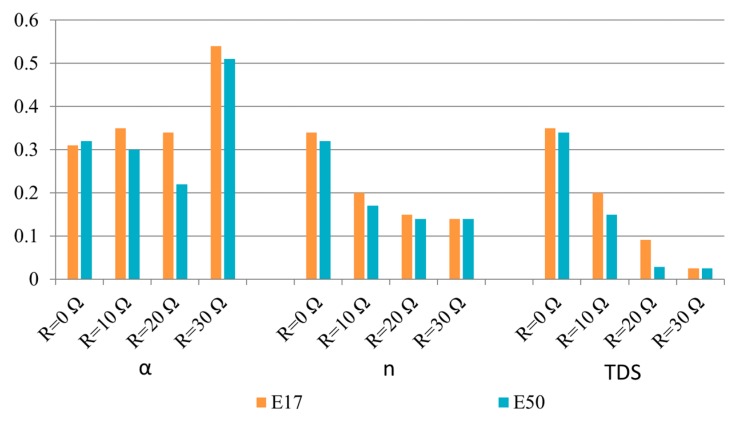
Primary smart sensor setting for a single-phase-to-ground fault under different DG penetrations.

A study to ascertain the impact of the PV penetration level as well as fault impedance on the protection coordination was performed for Scenario E (Table 2), corresponding to a single-phase-to-ground fault in the main feeder (line 6–7). In Scenario E, different fault resistance values of 0 Ω, 10 Ω, 20 Ω, and 30 Ω were considered. Moreover, both DG units presented in the smart grid (Figure 7) were in operation, and two different penetration levels were assessed: 17% (E17) and 50% (E50). Table 7 presents the operation time of the proposed smart-sensor protection coordination compared with the results obtained by classical coordination techniques with ER and linear programing-based techniques with IEDs. In all situations, the proposed APS running in the smart sensors’ MPU provides optimal setting parameters that reduce the tripping time of the smart sensors, as seen in Figure 10. Note that the proposed smart-sensor protection coordination changes the setting of the primary and backup smart sensors for any variation in the DG penetration or fault resistance value.

It must be highlighted that when the fault resistance and the DG penetration level increase, the classical (electromechanical relays) and linear schemes (IEDs) fail to isolate the fault. Furthermore, for the 50% DG penetration level and single-phase-to-ground fault with a fault resistance greater than zero, the smart-sensor protection coordination is the only scheme capable of protecting the network—by adapting the protection setting parameters to obtain a fast response—and maintaining the selectivity, as can be seen in Table 7.

## 6. Conclusions

The integration of distributed generation into smart grids could cause malfunctioning of the protection schemes, mainly because of the bidirectional power flows and their contribution to fault currents. With the aim of improving the reliability of smart grids, new protective devices and protection schemes must be developed to deal with the renewable energy impact on fault currents. The ability of smart sensors to acquire, monitor, and process measured data, as well as to execute optimization algorithms in their MPU, plays a vital role in smart grid protection. Incorporating smart sensors into the protection schemes reduces the activation time of network breakers and thus improves smart grid reliability.

In this paper, a relay-based smart sensor is proposed that offers the capability of updating its adjustment settings in real time in coordination with the BR. The protection scheme is able to optimize the setting parameters of the smart sensors for different network operation conditions. It employs three setting parameters (α, n, and TDS) in the optimization process, so that the operating area of the relay-based smart sensor is expanded, compared with the time-current curves defined by IEC 60255-151. The communication of the proposed smart-sensor protection coordination scheme can be implemented in a smart grid by using the communication standard IEC61850, so that smart sensors’ protection setting parameters as well as network breaker opening signals are exchanged via GOOSE messages.

The proposed relay-based smart sensor and the protection scheme were applied to a smart grid with DGs. The performance of the smart-sensor protection scheme was tested for different DG penetration levels, fault types (single-phase-to-ground and three-phase faults), and faulted zones (faults in the main feeder and in the laterals). In all cases, the APS hosted in the smart sensors’ MPU was capable of adapting, in real time, the performance parameters of the smart sensors responsible for protecting the faulted zone according to the operating conditions of the smart grid at the time of the fault measured by the smart sensors. Moreover, the adaptive smart-sensor protection coordination scheme proposed in this paper reduced the activation time of the network breakers by more than 80% and 64% compared with analogue electromechanical relays (classical coordination) and IEDs (linear programming), respectively. This ultimately translates into a more reliable network operation in the presence of DGs. Furthermore, it has been demonstrated that the relay-based smart sensor proposed in this paper is the only option that is able to clear the faulted zone of a smart grid with DGs when subjected to high impedance fault conditions. In this paper, it has been shown that information and communication technologies (ICT) play a vital role in smart grids where the IEC 61850 standard has started to be used for smart grid protection. One of the more important challenges is to integrate wireless communication into smart grid devices interconnecting an increasing number of smart grid sensors in the IoT world. In the future, the proposed smart sensor will be based on IoT-IEC 61850 communication offering wireless peer-to-peer communication between relay-based sensors (PR, BR) by means of GOOSE messages improving the communication among all the smart sensors spread over the grid. 

## Figures and Tables

**Table 1:** Parameters for different relay characteristics according to the IEC 60255-151 standard.

Time-Current Curve Type	Settings
Inverse	**α** = 0.14; n = 0.02
Very inverse	**α** = 13.5; n = 1
Extremely inverse	**α** = 80; n = 2

**Table 2:** GA chromosome structure.

Gen 1	Gen 2	Gen 3	...	Gen (3 * N_S_-2)	Gen 3 * (N_S_-1)	Gen (3 * N_S_)
$\alpha_{R1}$	$n_{R1}$	${\mathrm{TDS}}_{R1}$	…	$\alpha_{\mathrm{Rn}}$	$n_{\mathrm{Rn}}$	${\mathrm{TDS}}_{\mathrm{Rn}}\;$

**Table 3:** Zones of protection.

Zone	Buses		Lateral		Primary Relay-Based Smart Sensor	Backup Relay-Based Smart Sensor
	from	to	from	to		
1	1	4	4	20	S_1	-
2	4	8	8	22	S_2	S_1
3	8	12	12	23	S_3	S_2
4	12	14	14	25	S_4	S_3
5	14	16	16	26	S_5	S_4
6	16	19	-	-	S_6	S_5
7	-	-	27	30	S_7	S_6

**Table 4:** Scenarios.

Scenario	DG20	DG22	Fault		
			Type	Location	Line
A	✓	-	Three-phase	Main feeder	6–7
B	-	✓			
C	✓	✓			
D	✓	-	Single-phase-to ground	Main feeder	6–7
E	✓	✓			
F	✓	✓	Single-phase-to ground	Lateral	27–28

**Table 5:** APS optimal settings for S_1 and S_2 (Scenario B).

	α	n	TDS
S_1	0.33	0.33	0.36
S_2	2	0.14	0.025

**Table 6:** Comparison of operating times (classical, linear programming, and smart-sensor APS coordination) for different fault types and locations.

Scenario	T[s] (Classical)		T[s] (Linear)		T[s] (APS)	
	S_1	S_2	S_1	S_2	S_1	S_2
A	2.99	0.2	4.1	0.26	0.39	0.1
C	2.71	0.2	2.6	0.27	0.287	0.1
D	0.76	0.31	1.17	0.26	0.32	0.1
E	0.75	0.32	1.1	0.27	0.287	0.1
F	0.53	0.2	0.66	0.16	0.29	0.1

**Table 7:** Operation time comparison (classic, linear programming, and smart-sensor APS coordination) for different DG penetration levels: Scenario E.

		T[s] E17			T[s] E50		
		Classical	Linear	APS	Classical	Linear	APS
R = 0 [Ω]	S_1	0.7	1.09	0.3	0.75	1.1	0.29
	S_2	0.31	0.26	0.1	0.32	0.27	0.1
R = 10 [Ω]	S_1	2.13	2.32	0.3	-	-	0.29
	S_2	0.73	0.6	0.1	0.77	0.64	0.1
R = 20 [Ω]	S_1	-	-	0.29	-	-	0.31
	S_2	-	-	0.1	-	-	0.1
R = 30 [Ω]	S_1	-	-	0.32	-	-	0.34
	S_2	-	-	0.1	-	-	0.1

## Abbreviations

APS	adaptive protection scheme
BR	backup relay
CTI	coordination time interval
DG	distributed generation
ER	electromechanical relay
GA	genetic algorithm
GOOSE	generic object-oriented substation event
IED	intelligent electronic devices
MINLP	mixed-integer nonlinear programming
MPU	microprocessor unit
PMU	phase measurement units
PR	primary relay
PSM	plug setting multiplier
PV	photovoltaic
$T_{r}$	smart sensor operating time
TDS	time-dial setting
S_i	*i^th^* smart sensor

## References

[1] Jenkins N., Long C., Wu J. (2015). An overview of the smart grid in Great Britain. Engineering.

[2] Song E.Y., Fitz Patrick G.J., Lee K.B. (2017). Smart sensors and standard-based interoperability in smart grids. IEEE Sens. J..

[3] Delle Femine A., Gallo D., Landi C., Lo Schiavo A., Luiso M. (2019). Low power contactless voltage sensor for low voltage power systems. Sensors.

[4] Morello R., De Capua C., Fulco G., Mukhopadhyay S.C. (2017). A smart power meter to monitor energy flow in smart grids: The role of advanced sensing and IoT in the electric grid of the future. IEEE Sens. J..

[5] Islam T., Mukhopadhyay S.C., Suryadevara N.K. (2017). Smart sensors and internet of things: A postgraduate paper. IEEE Sens. J..

[6] Dileep G. (2020). A survey on smart grid technologies and applications. Renew. Energy.

[7] Rojas-Delgado B., Alonso M., Amaris H., de Santiago J. (2019). Wave power output smoothing through the use of a high-speed kinetic buffer. Energies.

[8] Alonso M., Amaris H., Germain J.G., Galan J.M. (2014). Optimal charging scheduling of electric vehicles in smart grids by heuristic algorithms. Energies.

[9] Vazquez R., Amaris H., Alonso M., Lopez G., Moreno J.I., Olmeda D., Coca J. (2017). Assessment of an adaptive load forecasting methodology in a smart grid demonstration project. Energies.

[10] (2007). IEEE Recommended Practice for the Design of Reliable Industrial and Commercial Power Systems.

[11] Shalash N.A., Ahmad A.Z. New reliability index for power system protection based on multi-agent technique. Proceedings of the 3rd International Conference on Electric Power and Energy Conversion Systems, EPECS 2013.

[12] Fuchs E.F., Masoum M.A.S. (2008). Power Quality in Power Systems and Electrical Machines.

[13] Norshahrani M., Mokhlis H., Abu Bakar A.H., Jamian J.J., Sukumar S. (2017). Progress on protection strategies to mitigate the impact of renewable distributed generation on distribution systems. Energies.

[14] Amaris H., Molina Y.P., Alonso M., Luyo J.E. (2018). Loss allocation in distribution networks based on Aumann–Shapley. IEEE Trans. Power Syst..

[15] Conde A., Vazquez E. (2011). Application of a proposed overcurrent relay in radial distribution networks. Electr. Power Syst. Res..

[16] Huchel Ł., Zeineldin H.H. (2016). Planning the coordination of directional overcurrent relays for distribution systems considering DG. IEEE Trans. Smart Grid.

[17] (2011). Roadmap for Moving to a Competitive Low Carbon Economy in 2050; Communication from the European Commission, “Energy Roadmap 2050”.

[18] Kezunovic M. (2011). Smart fault location for smart grids. IEEE Trans. Smart Grid.

[19] Eissa M.M., Awadalla H.A. (2019). Centralized protection scheme for smart grid integrated with multiple renewable resources using internet of energy. Glob. Transit..

[20] Silos A., Señís A., De Pozuelo R.M., Zaballos A. (2017). Using IEC 61850 goose service for adaptive ANSI 67/67N protection in ring main systems with distributed energy resources. Energies.

[21] Monadi M., Amin Zamani M., Candela J.I., Luna A., Rodriguez P. (2015). Protection of AC and DC distribution systems embedding distributed energy resources: A comparative review and analysis. Renew. Sustain. Energy Rev..

[22] (2016). Communication Networks and Systems in Substations.

[23] IEC (2009). Measuring Relays and Protection Equipment—Part 151: Functional Requirements for over/under Current Protection.

[24] Anderson P.M. (1998). Power System Protection.

[25] Tleis N. (2019). Power Systems Modelling and Fault Analysis, Theory and Practice.

[26] (2018). IEEE Standard for Interconnection and Interoperability of Distributed Energy Resources with Associated Electric Power Systems Interfaces.

[27] Hooshyar H., Baran M.E. (2013). Fault analysis on distribution feeders with high penetration of PV systems. IEEE Trans. Power Syst..

[28] Al-Shetwi A.Q., Sujod M.Z., Ramli N.L. (2015). A review of the fault ride through requirements in different grid codes concerning penetration of PV system to the electric power network. ARPN J. Eng. Appl. Sci..

[29] Padullaparti H.V., Chirapongsananurak P., Hernandez M.E., Santoso S. (2016). Analytical approach to estimate feeder accommodation limits based on protection criteria. IEEE Access.

[30] (2014). IEEE Guide for Conducting Distribution Impact Studies for Distributed Resource Interconnection.

[31] Papaspiliotopoulos V.A., Kleftakis V.A., Kotsampopoulos P.C., Korres G.N., Hatziargyriou N.D. (2012). Hardware-in-the-loop simulation of protection blinding and sympathetic tripping phenomena in modern distribution grids. Proc. Energy.

[32] (2007). IEEE Guide for Protective Relay Applications to Distribution Lines.

[33] Sung B.C., Lee S.H., Park J., Meliopoulos A.P.S. (2013). Adaptive protection algorithm for overcurrent relay in distribution system with DG. J. Electr. Eng. Technol..

[34] Zeineldin H.H., Sharaf H.M., Ibrahim D.K., El-Zahab E.E.D.A. (2015). Optimal protection coordination for meshed distribution systems with DG using dual setting directional over-current relays. IEEE Trans. Smart Grid.

[35] Kumar D.S., Srinivasan D., Reindl T. (2016). A fast and scalable protection scheme for distribution networks with distributed generation. IEEE Trans. Power Deliv..

[36] Coffele F., Booth C., Dyśko A. (2015). An adaptive overcurrent protection scheme for distribution networks. IEEE Trans. Power Deliv..

[37] Kayastha N., Niyato D., Hossain E., Han Z. (2014). Smart grid sensor data collection, communication, and networking: A tutorial. Wirel. Commun. Mob. Comput..

[38] Hojabri M., Dersch U., Papaemmanouil A., Bosshart P. (2019). A comprehensive survey on phasor measurement unit applications in distribution systems. Energies.

[39] Phadke A.G., Bi T. (2018). Phasor measurement units, WAMS, and their applications in protection and control of power systems. J. Mod. Power Syst. Clean Energy.

[40] Sanduleac M., Lipari G., Monti A., Voulkidis A., Zanetto G., Corsi A., Toma L., Fiorentino G., Federenciuc D. (2017). Next generation real-time smart meters for ICT based assessment of grid data inconsistencies. Energies.

[41] Perez L.G., Urdaneta A.J. (2001). Optimal computation of distance relays second zone timing in a mixed protection scheme with directional overcurrent relays. IEEE Trans. Power Deliv..

[42] Chattopadhyay B., Sachdev M.S., Sidhu T.S. (1996). An on-line relay coordination algorithm for adaptive protection using linear programming technique. IEEE Trans. Power Deliv..

[43] Laway N.A., Gupta H.O. A method for adaptive coordination of overcurrent relays in an interconnected power system. Proceedings of the 5th International Conference on Developments in Power System Protection.

[44] González D.A. (2016). Coordinación De Protecciones En Redes Eléctricas Con Generación Distribuida. Ph.D. Thesis.

[45] Thangaraj R., Pant M., Deep K. (2010). Optimal coordination of over-current relays using modified differential evolution algorithms. Eng. Appl. Artif. Intell..

[46] Sueiro J.A., Diaz-Dorado E., Míguez E., Cidrás J. (2012). Coordination of directional overcurrent relay using evolutionary algorithm and linear programming. Int. J. Electr. Power Energy Syst..

[47] Xu K., Liao Y. Intelligent method for online adaptive optimum coordination of overcurrent relays. Proceedings of the Clemson University Power Systems Conference (PSC2018).

[48] Rezaei N., Uddin M.N., Amin I.K., Othman M.L., Marsadek M. (2019). Genetic algorithm-based optimization of overcurrent relay coordination for improved protection of DFIG operated wind farms. IEEE Trans. Ind. Appl..

[49] Solati Alkaran D., Vatani M.R., Sanjari M.J., Gharehpetian G.B., Naderi M.S. (2018). Optimal overcurrent relay coordination in interconnected networks by using fuzzy-based GA method. IEEE Trans. Smart Grid.

[50] Thakur M., Meghwani S.S., Jalota H. (2014). A modified real coded genetic algorithm for constrained optimization. Appl. Math. Comput..

[51] Rawat R., Kale V.S., Gokhale S.S. Application of nature inspired metaheuristic techniques to overcurrent relay coordination. Proceedings of the Future Technologies Conference (FTC).

[52] So P., Li K.K. (2000). Time coordination method for power system protection by evolutionary algorithm. IEEE Trans. Ind. Appl..

[53] Xu S.-H., Liu J.-P., Zhang F.-H., Wang L., Sun L.-J. (2015). A combination of genetic algorithm and particle swarm optimization for vehicle routing problem with time windows. Sensors.

[54] Kersting W.H. Radial Distribution Test Feeders. Proceedings of the IEEE Power Eng. Soc. Winter Meeting.

[55] Shih M.Y., Conde A., Angeles-Camacho C. (2019). Enhanced self-adaptive differential evolution multi-objective algorithm for coordination of directional overcurrent relays contemplating maximum and minimum fault points. IET Gener. Transm. Distrib..

[56] MATLAB (2017). 9.3.0.1190202 (R2017b).

[57] Polajžer B., Pintarič M., Rošer M., Štumberger G. (2019). Protection of MV closed-loop distribution networks with Bi-directional overcurrent relays and goose communications. IEEE Access.

[58] Ito H., Ohashi K. (2008). High Performance IEC 61850 GOOSE and Protection Relay Testing; PAC. https://www.pacw.org/no-cache/issue/winter_2008_issue/protection_goose/high_performance_iec_61850_goose_and_protection_relay_testing.html.

[59] Bayliss C. (2006). Transmission and Distribution Electrical Engineering: Electrical Engineering.

